# Supplementary material for: Targeting the transmembrane cytokine co-receptor neuropilin-1 in distal tubules improves renal injury and fibrosis
Source: Nat Commun. 2024 Jul 8;15:5731. doi: 10.1038/s41467-024-50121-6 (PMC11231174; doi:10.1038/s41467-024-50121-6)
Supplement: Supplementary file 1 — Supplementary Information [file 41467_2024_50121_MOESM1_ESM.pdf]

SUPPLEMENTARY INFORMATION

Title:

Targeting the transmembrane cytokine co-receptor neuropilin-1 in distal tubules improves renal injury and fibrosis

Author list:

Yinzheng Li[1], Zheng Wang[1], Huzi Xu[1], Yu Hong[1], Mengxia Shi[1], Bin Hu[1], Xiuru Wang[1], Shulin Ma[1], Meng Wang[1], Chujin Cao[1], Han Zhu[1], Danni Hu[1], Chang xu[1], Yanping Lin[1], Gang Xu[1,*], Ying Yao[1,2,*], Rui Zeng[1,3,*]

Affiliations:

[1] Division of Nephrology, Tongji Hospital, Tongji Medical College, Huazhong University of Science and Technology, 1095 Jiefang Ave, Wuhan 430030, China

[2] Department of Nutrition, Tongji Hospital, Tongji Medical College, Huazhong University of Science and Technology, 1095 Jiefang Ave, Wuhan 430030, China

[3] Key Laboratory of Organ Transplantation, Ministry of Education, NHC Key Laboratory of Organ Transplantation, Key Laboratory of Organ Transplantation, Chinese Academy of Medical Sciences, Wuhan 430030, China.

*Correspondence: zengrui@tjh.tjmu.edu.cn (R.Z.), yaoyingkk@126.com (Y.Y.), xugang@tjh.tjmu.edu.cn (G.X.)

Inventory of Supporting Information:

Supplementary Figures 1 to 10

Supplementary Tables 1 to 3

References 1 to 3

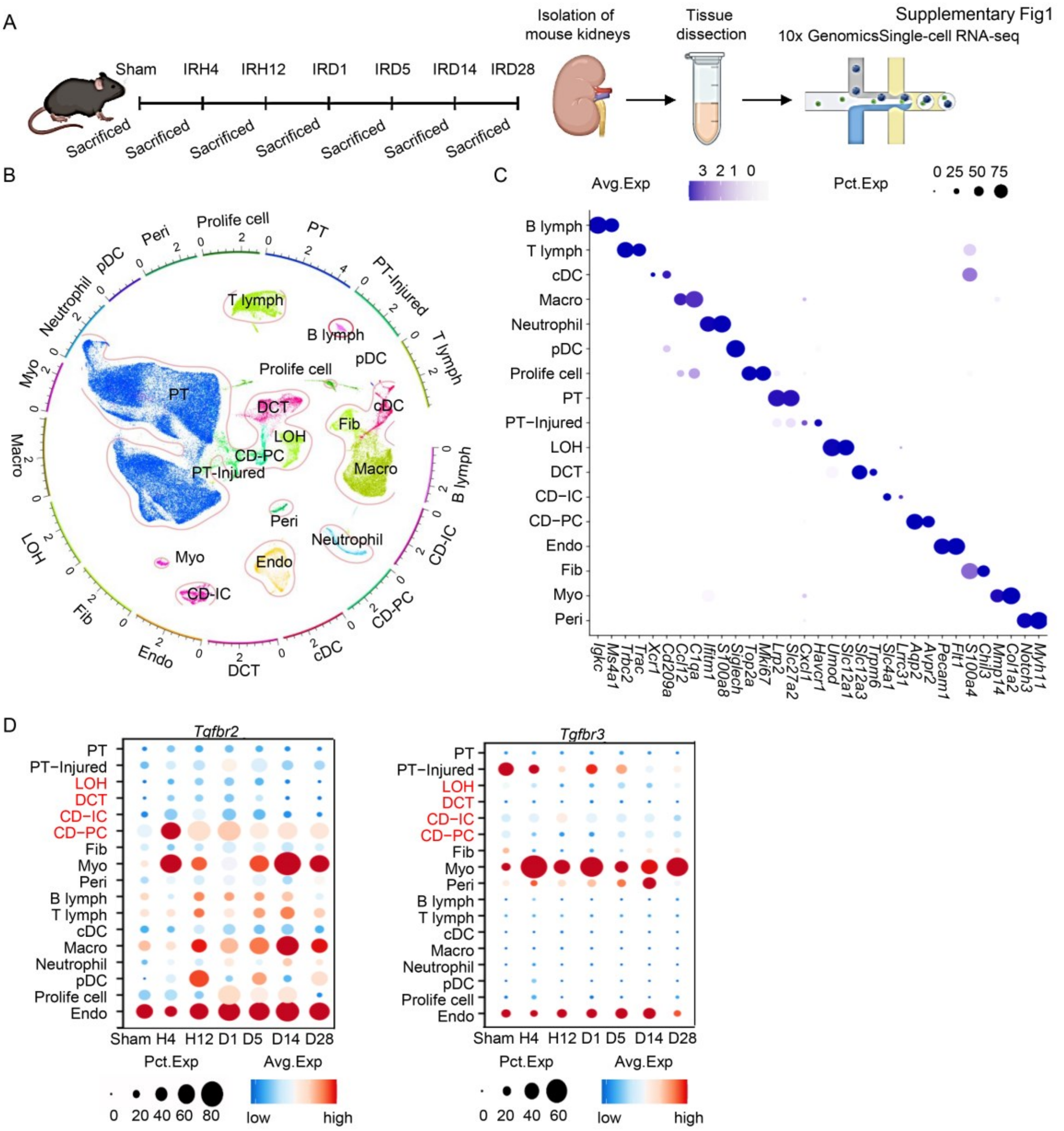

Supplementary Figure 1. A single-cell transcriptomics landscape of mouse kidney after I-R. (A) A schematic diagram illustrating the workflow for single-cell RNA sequencing sample submission. Supplementary Figure 1A, created with BioRender.com, released under a Creative Commons Attribution-NonCommercial-NoDerivs 4.0 International license. (B) A UMAP presentation depicted cells profiled from individual mouse kidneys under healthy or IR conditions. The surrounding circular layouts represent the cell number of each population on a logarithmic scale. PT, proximal tubule; PT-Injured, injured PT; LOH, loop of Henle ; DCT, distal convoluted tubule; CD-PC, principal cell of collecting duct; CD-IC,

intercalated cell of collecting duct; EC, endothelial cell; Peri, Pericytes; Fib, fibroblast; Myo, myofibroblast; Prolife cell, proliferating cell; Macro, macrophage; T lymph, T lymphocyte; B lymph, B lymphocyte; cDC, conventional dendritic cell; pDC, plasmacytoid dendritic cell; Neutrophil, Neutrophil. (C) Dotplot showing gene expression levels and proportions of cluster-specific marker genes in each cluster. (D) Expression levels of *Tgfbr2* and *Tgfbr3* in kidney after IR surgery.

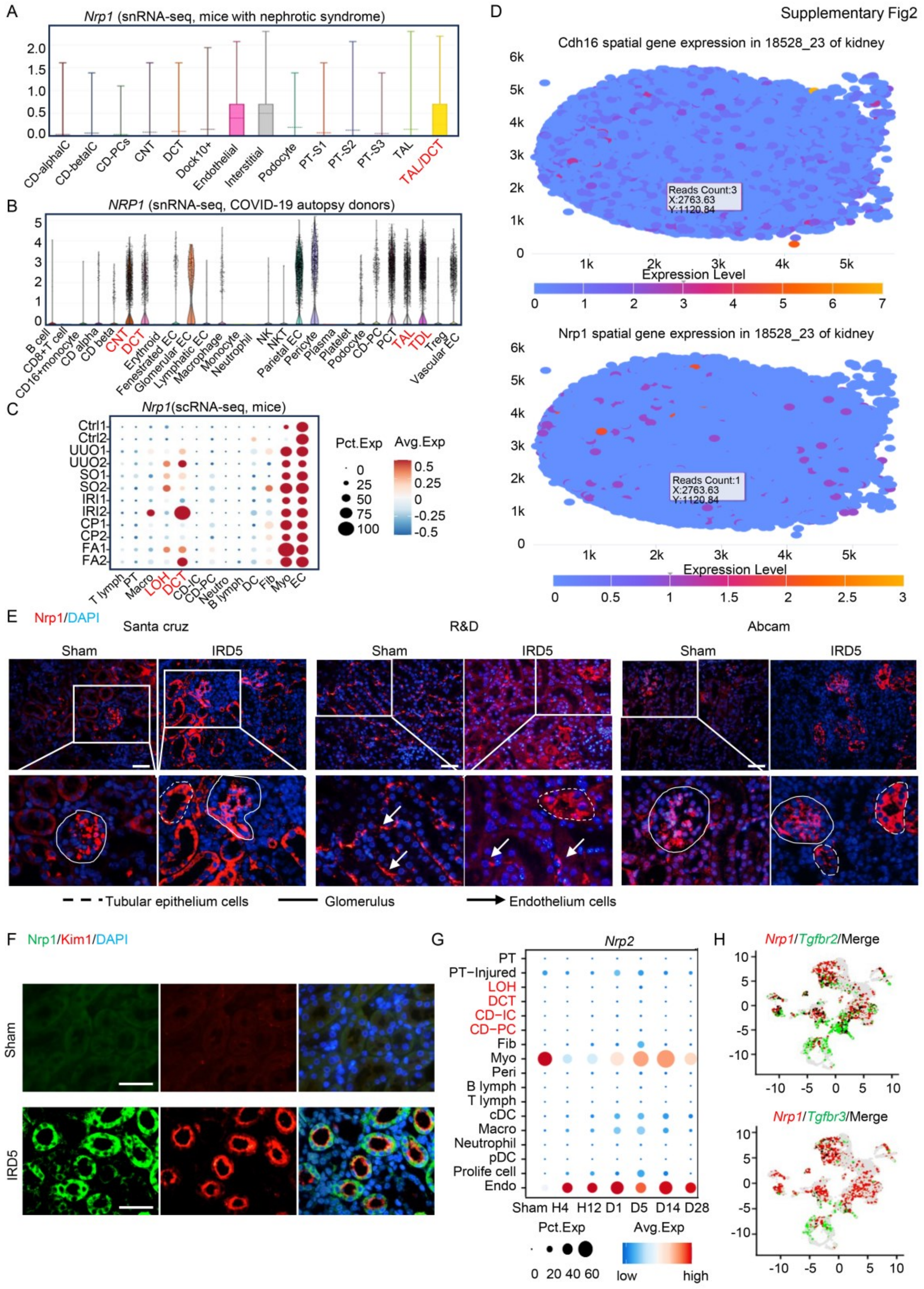

Supplementary Figure 2. Nrp1 is expressed in distal TECs. (A) Expression levels of *Nrp1* in mice with nephrotic syndrome. Figure created with https://singlecell.broadinstitute.org/. The data is pubic in Single Cell Portal whitepaper

(https://www.biorxiv.org/content/10.1101/2023.07.13.548886v1). (B) Expression of *Nrp1* in COVID-19 autopsy donors. Image from https://singlecell.broadinstitute.org/. The data is pubic in Single Cell Portal whitepaper (https://www.biorxiv.org/content/10.1101/2023.07.13.548886v1). (C) The expression levels of *Nrp1* in the mice kidneys after UUO, sodium oxalate (SO), IR, cisplatin (CP), and folic acid (FA). The data comes from GSE197266. (D) Expression of Nrp1 and Cdh16 in mouse renal space transcriptomics. Image from https://www.spatialomics.org/SpatialDB/. (E) Nrp1 immunofluorescence staining with antibody from Santa cruz, R&D and Abcam. (F) Co-expression of Nrp1 with Kim1 with immunofluorescence staining. (G) Expression levels of *Nrp2* in kidney after IR surgery in our study. (H) Co-expression of Nrp1 and TGF-β receptors in DT cells.

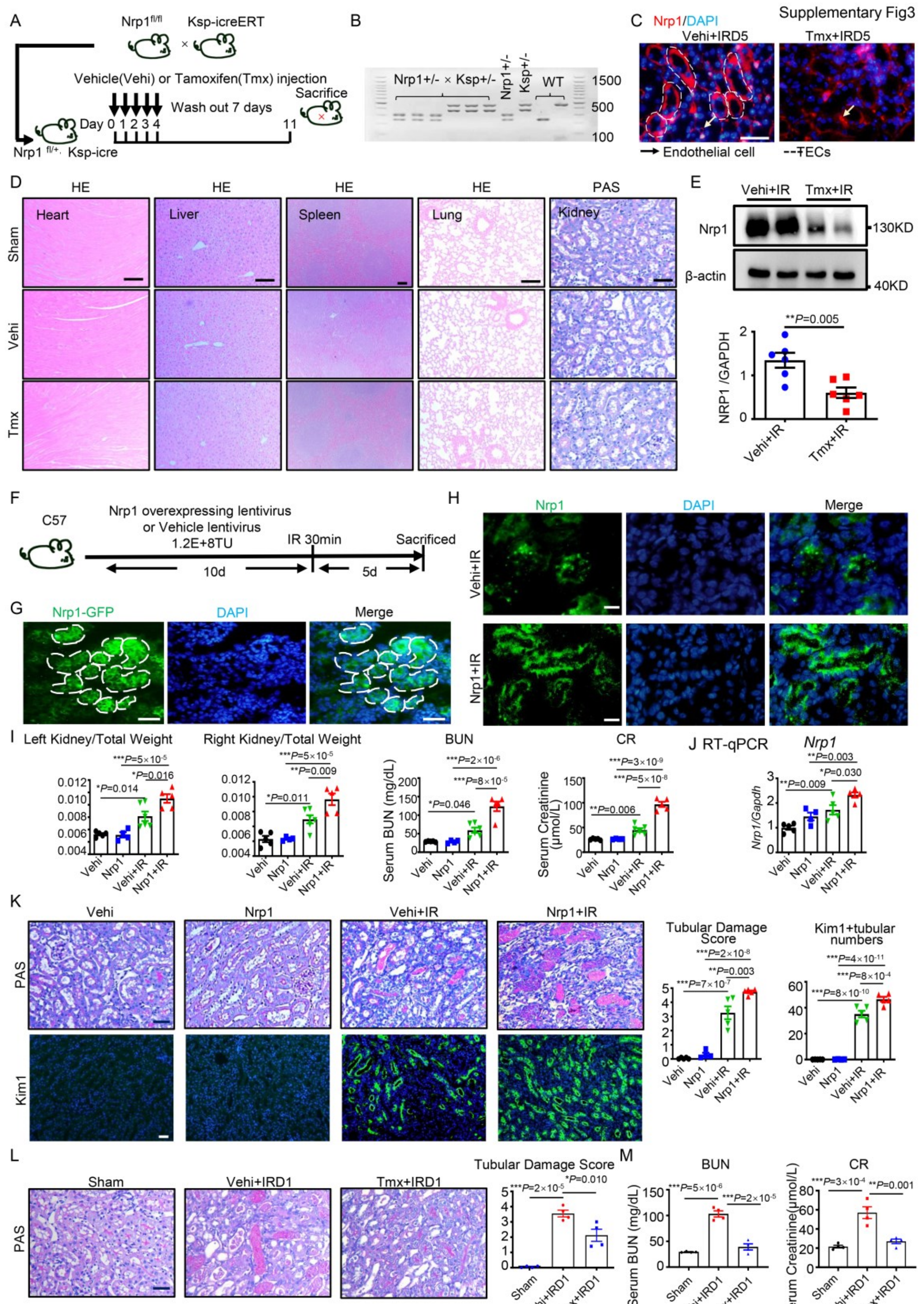

Supplementary Figure 3. Knockout or overexpression of *Nrp1* in distal TECs. (A) A schematic diagram illustrating tubular *Nrp1* deletion. (B) Mouse tail DNA analysis to validate gene knockouts. (C) Nrp1 immunofluorescence staining after IR in mice. (D)

Histological analyses of tissues from the heart, liver, spleen, lungs, and kidneys of the gene knockout mice by H&E and PAS staining. Scale bar, 50 μm. (E) Validation of Nrp1 expression in gene knockout by Western Blotting (n = 6 per group). (F) A schematic diagram illustrating the subcapsular injection of lentivirus for Nrp1 overexpression. (G) Fluorescence images of Nrp1 lentivirus with GFP. Scale bar, 20 μm. (H) Fluorescence images of Nrp1 on day 5 after I-R in mice. Scale bar, 20 μm. (I) Ratio of kidney weight versus body weight of mice (For Vehi, n=5; Nrp1, n=4; Vehi+IR, n=6; Nrp1+IR, n=5). Plasma BUN concentrations and creatinine (CR) concentrations in groups (For Vehi, n=5; Nrp1, n=4; Vehi+IR, n=6; Nrp1+IR, n=5). (J) Levels of mRNA encoding *Nrp1* by RT-qPCR (For Vehi, n=5; Nrp1, n=4; Vehi+IR, n=6; Nrp1+IR, n=5). (K) Representative micrographs and corresponding statistical scores of periodic acid-Schiff (PAS) staining and Kim1 immunofluorescence staining on day 5 after IR in mice (For Vehi, n=5; Nrp1, n=4; Vehi+IR, n=6; Nrp1+IR, n=5). (L) Representative micrographs and corresponding statistical scores of periodic acid-Schiff (PAS) staining on day 1 after IR in mice (n = 4 per group). (M) Plasma BUN concentrations and creatinine (CR) concentrations in groups on day 1 after IR in mice (n = 4 per group). Scale bar, 20 μm. *$P < 0.05$, **$P < 0.01$, ***$P < 0.001$ as determined by one-way ANOVA. Data represent mean ± SEM. Source data are provided as a Source Data file.

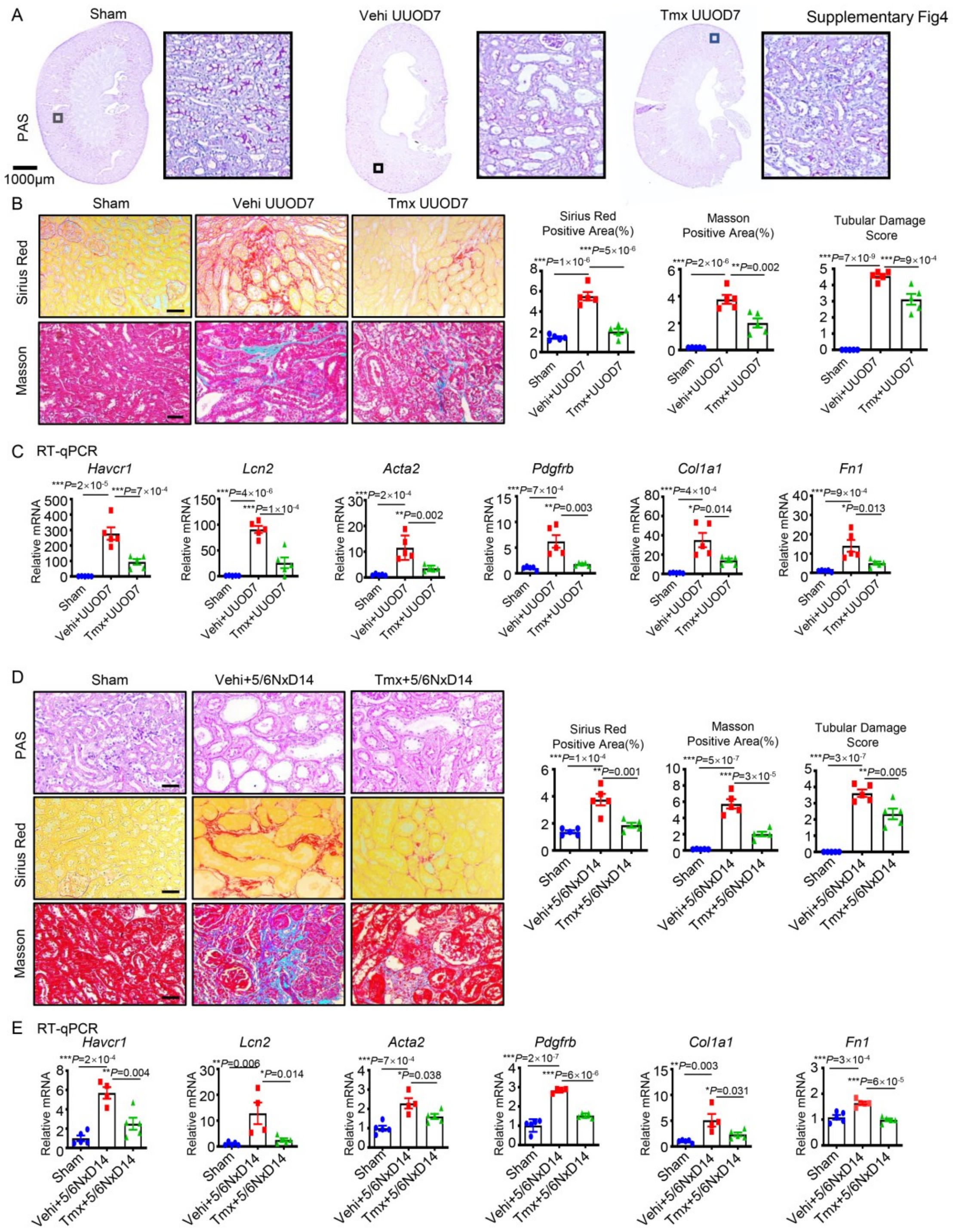

Supplementary Figure 4. Knockout of *Nrp1* in distal TECs alleviates renal fibrosis caused by UUO and 5/6 nephrectomy. (A) Whole-slide image of mouse kidney paraffin sections stained with PAS after UUO surgery and statistical analysis of renal tubular injury scores (n = 5 per group). (B) Representative micrographs and corresponding statistical scores of Sirius red and Masson on day 7 after UUO in mice (n = 5 per group). (C)

Expression levels of kidney damage related indicators (*Havcr1* and *Lcn2*) and fibrosis related indicators (*Acta2*, *Pdgfrb*, *Col1a1* and *Fn1*) at day 7 after UUO determined using RT-qPCR (n = 5 per group). (D) Representative micrographs and corresponding statistical scores of PAS, Sirius red and Masson on day 14 after 5/6 nephrectomy in mice (n = 5 per group). (E) Expression levels of kidney damage related indicators (*Havcr1* and *Lcn2*) and fibrosis related indicators (*Acta2*, *Pdgfrb*, *Col1a1* and *Fn1*) at day 14 after 5/6 nephrectomy determined using RT-qPCR (For Sham, n=5; Vehi+5/6NxD14, n=4; Tmx+5/6NxD14, n=5). *$P < 0.05$, **$P < 0.01$, ***$P < 0.001$ as determined by one-way ANOVA. Scale bar, 20 μm. Data represent mean ± SEM. Source data are provided as a Source Data file.

Supplementary Fig5

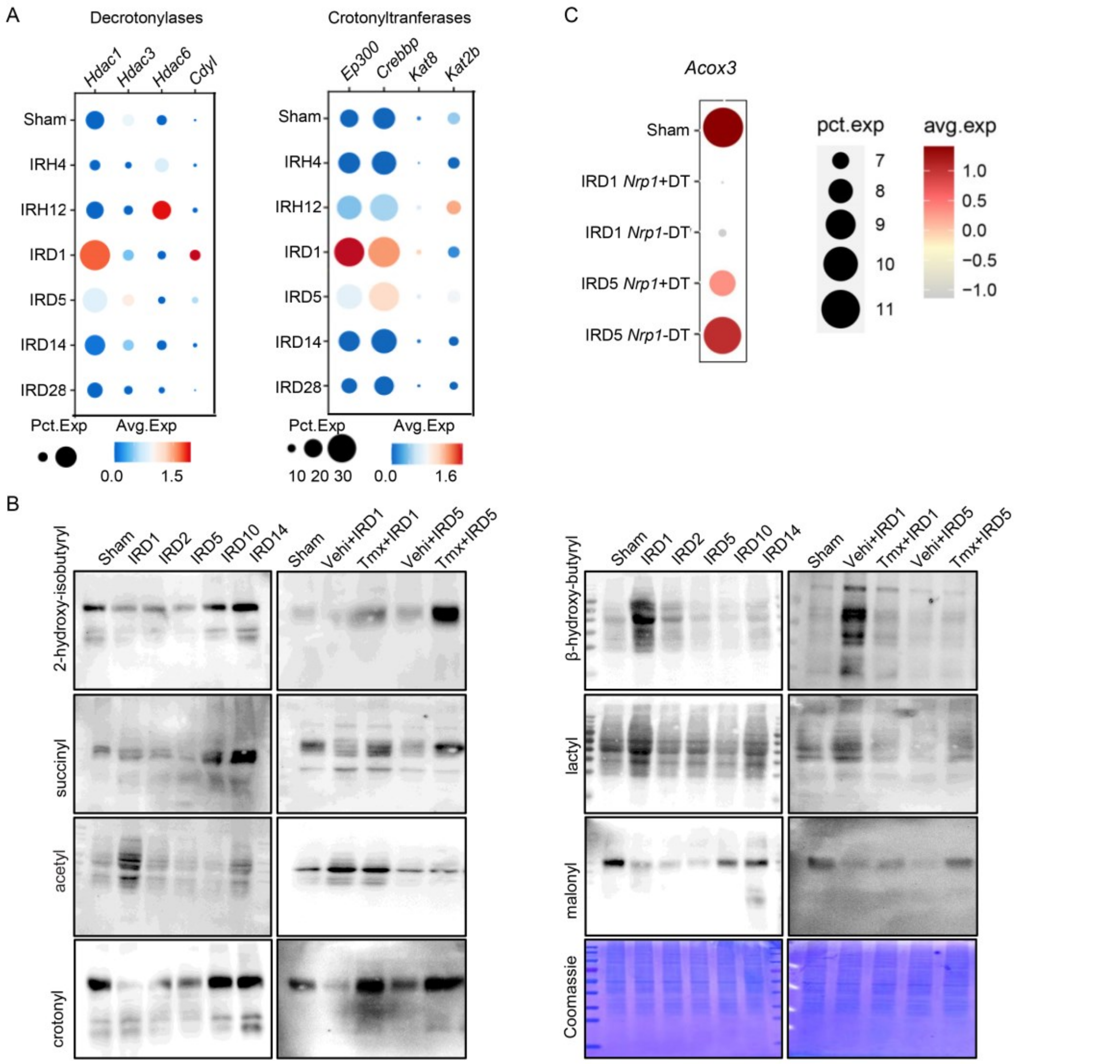

Supplementary Figure 5. Changes in enzyme regulating lysine modifications and other classical post-translational modifications after I-R-induced injury. (A) Expression levels of decrotonylases and crotonyltranferases genes in kidney after I-R surgery. (B) Western blotting analysis showing the changes in different lysine modifications after I-R and *Nrp1* knockout. (C) Expression levels of *Acox3* in kidney after I-R surgery. Source data are provided as a Source Data file.

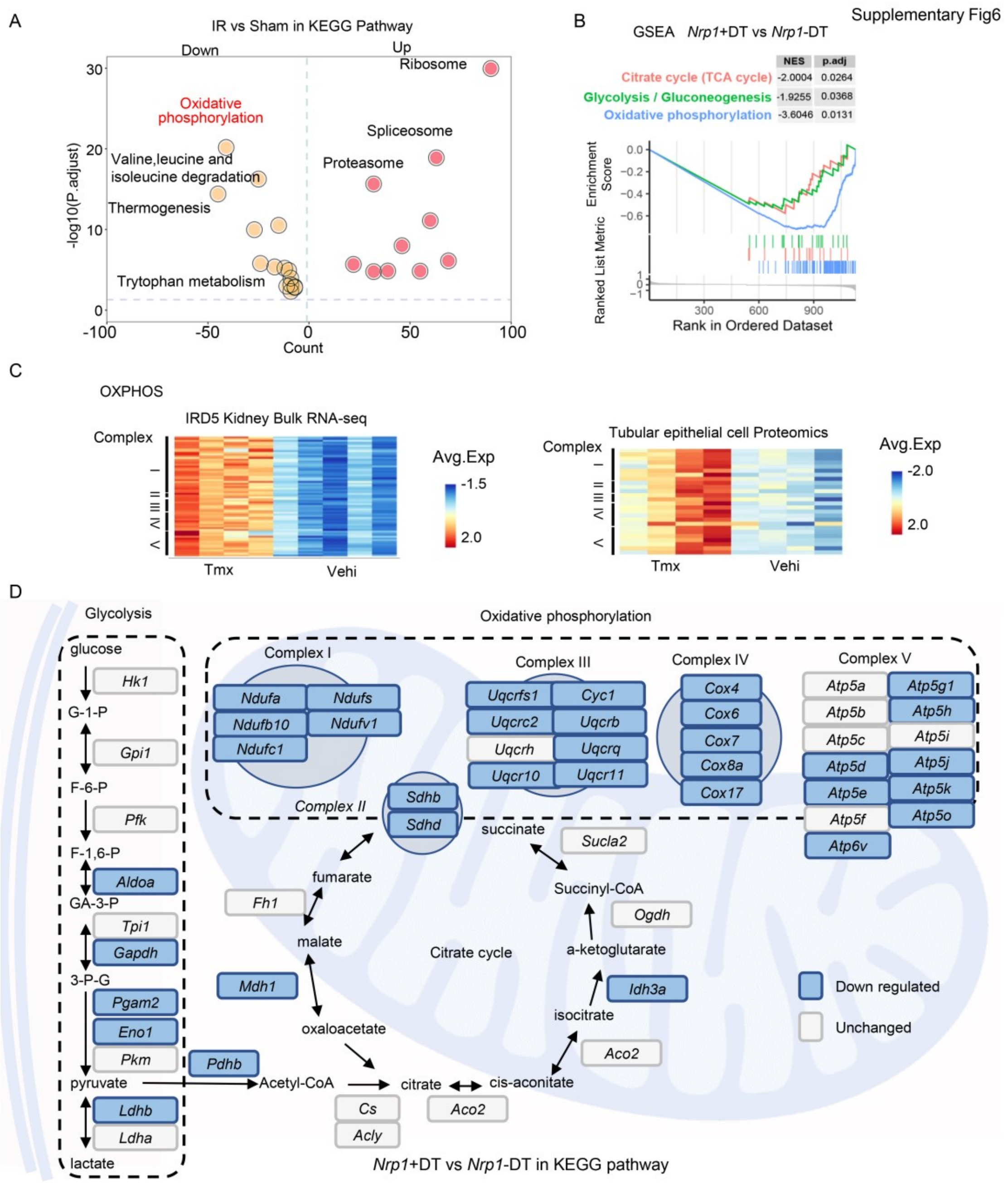

Supplementary Figure 6. Nrp1 inhibits OXPHOS levels in distal TECs. (A) Regulated KEGG pathways in I-R groups compared to sham groups in scRNA-seq data. The horizontal axis represents the number of genes with significant differences enriched in different pathways, positive values represent the number of genes upregulated by I-R compared to the sham group, and negative values represent the number of genes downregulated by I-R compared to the sham group. (B) Gene Set Enrichment Analysis

(GSEA) plot depicting the enrichment of gene sets related to OXPHOS, TCA, and glycolysis/gluconeogenesis pathways in *Nrp1*+ DT cells compared to *Nrp1*- DT cells. (C) The OXPHOS-related heatmap in IRD5 kidney bulk RNA-seq and pTECs proteomics (n = 4 per group). (D) After IRD5, *Nrp1*+DT compared with *Nrp1*-DT, the pathway map of gene transcription level changes in OXPHOS, TCA and glycolysis pathway in scRNA-seq data. Scale: Blue corresponds to gene downregulation in *Nrp1*+DT compared to *Nrp1*- DT, while gray indicates no statistically significant change in gene expression. Source data are provided as a Source Data file.

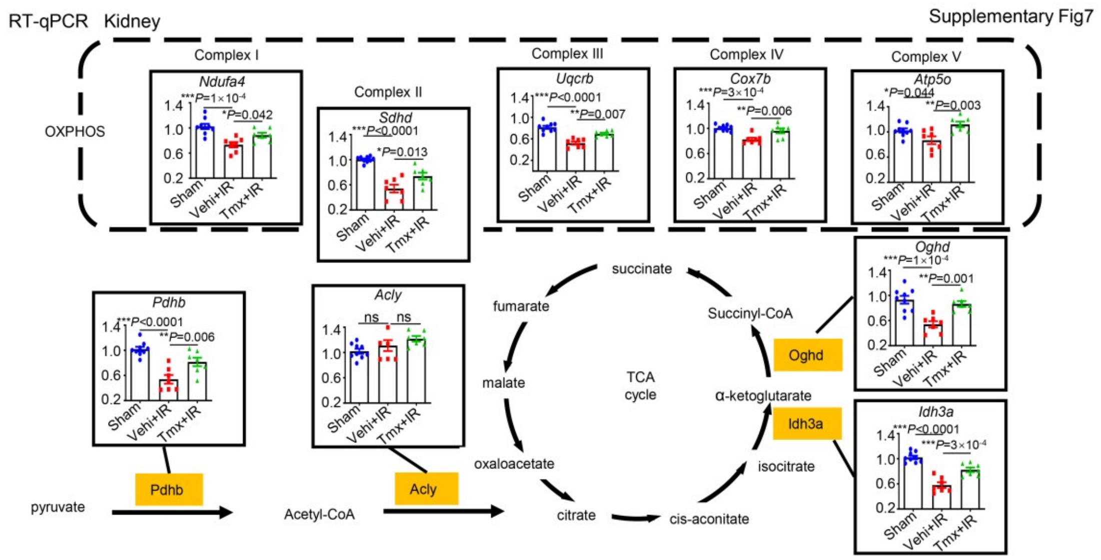

Supplementary Figure 7. Nrp1 inhibited OXPHOS levels. Renal tissue expression of OXPHOS and TCA at day 5 after IR determined using RT-qPCR (For Sham, *n*=9; Vehi+IR, *n*=7; Tmx+IR, *n*=7). **P* < 0.05, ***P* < 0.01, ****P* < 0.001 as determined by one-way ANOVA. Data represent mean ± SEM. Source data are provided as a Source Data file.

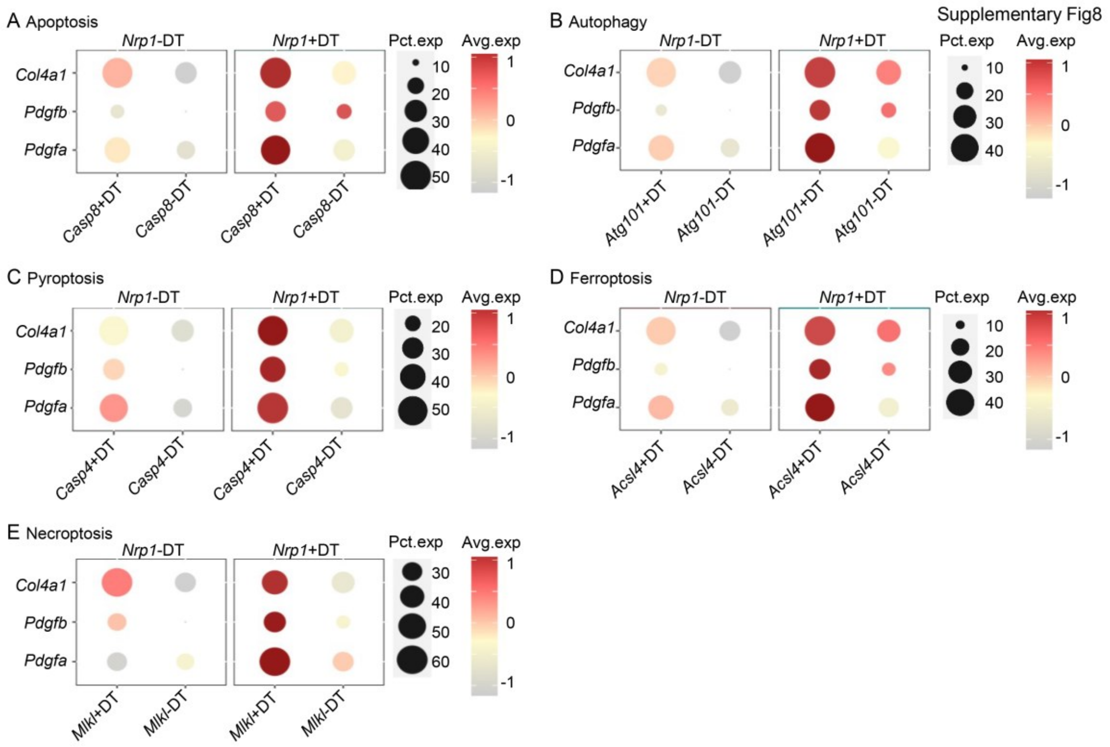

Supplementary Figure 8. *Nrp1*+DT cells are rich in expression of cell death-related genes and secrete large amounts of pro-fibrotic factors. (A) Dotplot of *Col4a1*, *Pdgfb*, and *Pdgfa* expression in DT cells expressing the apoptosis representative gene *Casp8*. (B) Dotplot of *Col4a1*, *Pdgfb*, and *Pdgfa* expression in DT cells expressing the autophagy representative gene *Atg101*. (C) Dotplot of *Col4a1*, *Pdgfb*, and *Pdgfa* expression in DT cells expressing the pyroptosis representative gene *Casp4*. (D) Dotplot of *Col4a1*, *Pdgfb*, and *Pdgfa* expression in DT cells expressing the ferroptosis representative gene *Acsl4*. (E) Dotplot of *Col4a1*, *Pdgfb*, and *Pdgfa* expression in DT cells expressing the necroptosis representative gene *Mlkl*.

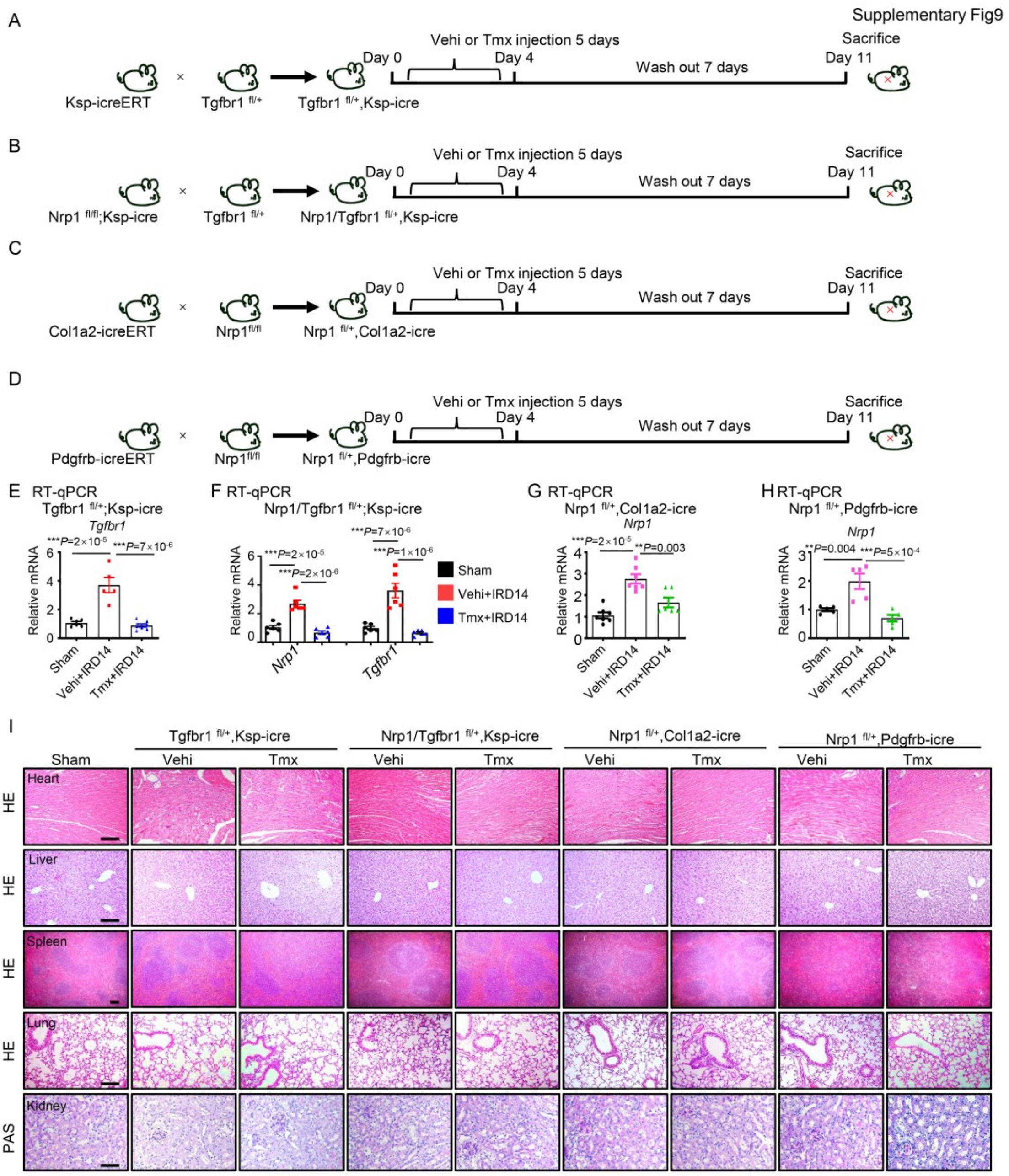

Supplementary Figure 9. Construction of distal tubular-specific *Nrp1* and *Tgfbr1* double-gene knockout mice, as well as myofibroblast-specific *Nrp1* knockout mice and pericyte-specific *Nrp1* knockout mice. (A) A schematic diagram illustrating the tubular-specific *Tgfbr1* knockout. (B) A schematic diagram illustrating the tubular-specific *Nrp1* and *Tgfbr1* double-gene knockout. (C) A schematic diagram illustrating the myofibroblast-specific *Nrp1* knockout. (D) A schematic diagram illustrating the pericyte-

specific *Nrp1* knockout. (E) RT-qPCR experiments were conducted using mice from A to determine the expression levels of *Nrp1* and/or *Tgfbr1* (For Sham, *n*=6; Vehi+IRD14, *n*=5; Tmx+IRD14, *n*=7). (F) RT-qPCR experiments were conducted using mice from B to determine the expression levels of *Nrp1* and/or *Tgfbr1* (*n* = 6 per group). (G) RT-qPCR experiments were conducted using mice from C to determine the expression levels of *Nrp1* and/or *Tgfbr1* (For Sham, *n*=7; Vehi+IRD14, *n*=7; Tmx+IRD14, *n*=6). (H) RT-qPCR experiments were conducted using mice from D to determine the expression levels of *Nrp1* and/or *Tgfbr1* (*n* = 5 per group). (I) Histological analyses of section from the heart, liver, spleen, lungs, and kidneys of the gene knockdown mice by H&E and PAS staining. *P < 0.05, **P < 0.01, ***P < 0.001 as determined by one-way ANOVA. Scale bar, 50 μm. Data represent mean ± SEM. Source data are provided as a Source Data file.

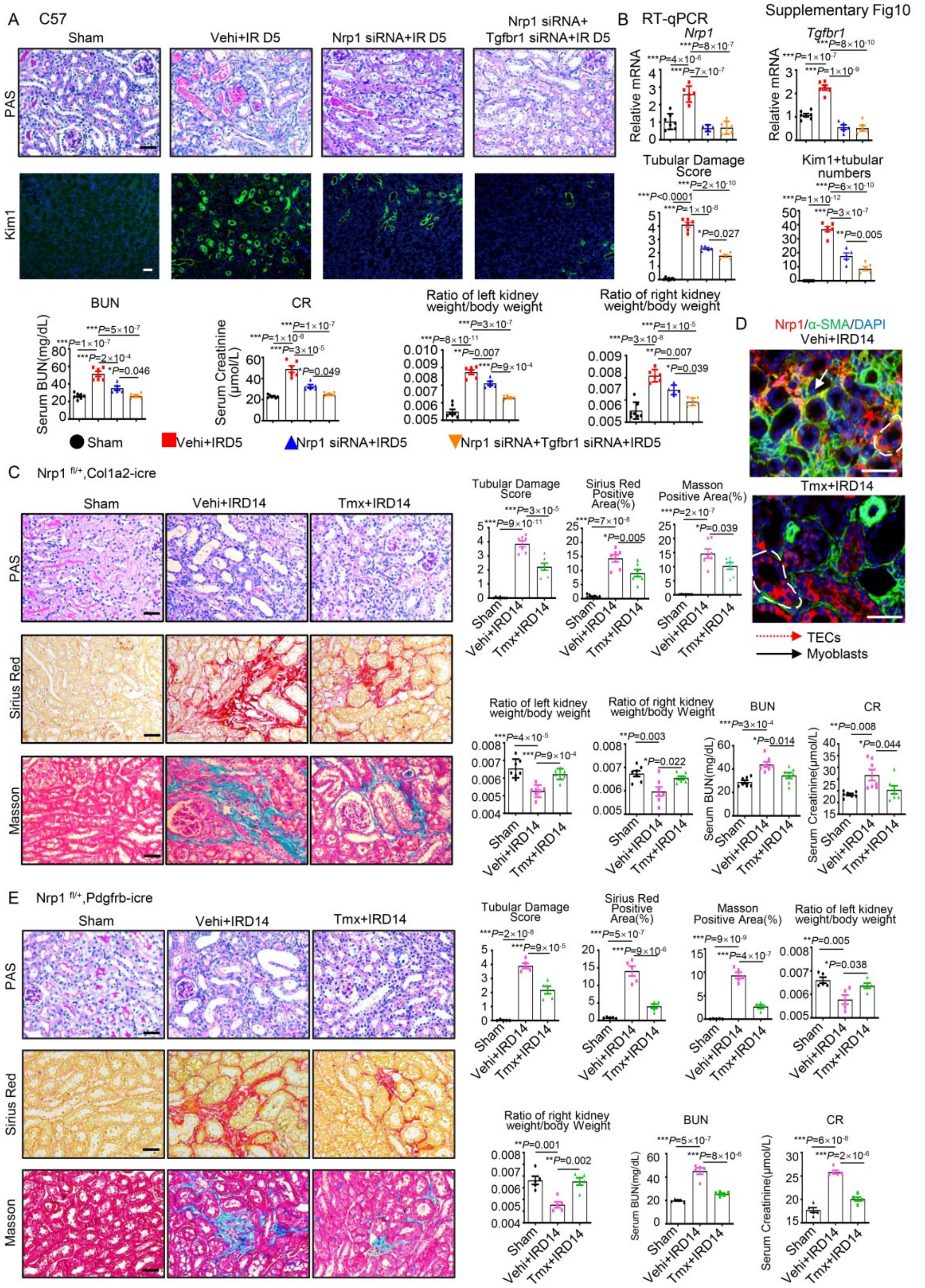

Supplementary Figure 10. Injection of *Nrp1* and *Tgfr1* siRNA under the renal capsule, or deletion of *Nrp1* in myofibroblasts or pericytes attenuates I-R-induced renal injury.

(A) Representative micrographs and corresponding statistical scores of PAS and Kim1

immunofluorescence staining on day 5 after renal capsule injection of Nrp1 and Tgfr1 siRNA in C57 mice with I-R injury. Ratio of kidney weight versus body weight of mice. Plasma BUN concentrations and CR concentrations in sham, Vehi + IR, Nrp1 siRNA + IR, or Nrp1 siRNA + Tgfr1 siRNA + IR groups at 5 days. For Sham, *n*=7; Vehi+IRD5, *n*=6; Nrp1 siRNA+IRD5, *n*=5; Nrp1 siRNA+Tgfbr1 siRNA+IRD5, *n*=5. (B) Expression levels of *Nrp1* and *Tgfbr1* in kidneys of C57 mice with IR injury, after renal capsule injection of Nrp1 and Tgfr1 siRNA, were detected by RT-qPCR (For Sham, *n*=7; Vehi+IRD5, *n*=6; Nrp1 siRNA+IRD5, *n*=5; Nrp1 siRNA+Tgfbr1 siRNA+IRD5, *n*=5). (C) Representative micrographs and corresponding statistical scores of PAS, Masson, and Sirius red staining on day 14 after I-R in mice with myofibroblast-specific *Nrp1* knockout (*n* = 7 per group). Ratio of kidney weight versus body weight of mice (*n* = 7 per group). Plasma BUN concentrations and CR concentrations in sham, Vehi + IR, or Tmx + IR groups at 14 days (*n* = 7 per group). (D) Co-expression of Nrp1 with α-SMA with immunofluorescence staining. (E) Representative micrographs and corresponding statistical scores of PAS, Masson, and Sirius red staining on day 14 after I-R in mice with pericyte-specific *Nrp1* knockout (*n* = 5 per group). Ratio of kidney weight versus body weight of mice (*n* = 5 per group). Plasma BUN concentrations and CR concentrations in sham, Vehi + IR, or Tmx + IR groups at 14 days (*n* = 5 per group). **P* < 0.05, ***P* < 0.01, ****P* < 0.001 as determined by one-way ANOVA. Scale bar, 20 μm. Data represent mean ± SEM. Source data are provided as a Source Data file.

Table S1. General characteristics of study participants (Related to Figure 1)

Variable	eGFR < 30 ml/min/1.73 m^2 (n = 115)	eGFR ≥ 30 ml/min/1.73 m^2 (n = 102)	*P*
Gender male	86/115(74.8%)	74/102(72.5%)	0.709[c]
Age (years)	41.72±1.039	40.11±1.14	0.296[a]
Hypertension positive	100/115(87.0%)	82/102(80.4%)	0.189[c]
Diabetes positive	11/115(9.6%)	11/102(10.8%)	0.767[c]
White blood cell,10^9/L	6.5±0.27	7.0±0.24	0.139[a]
Neutrophil,10^9/L	4.66±0.25	4.56±0.22	0.765[a]
Lymphocyte,10^9/L	1.37±0.16	1.78±0.07	0.031[a]
Monocyte,10^9/L	0.55±0.04	0.57±0.22	0.561[a]
Platelet,10^9/L	191.08±6.00	202.67±6.01	0.176[a]
Na^+,mmol/L	139.78±0.29	140.38±0.19	0.099[a]
K^+,mmol/L	7.27±2.76	4.32±0.06	0.321[a]
Cl^-,mmol/L	105.2(101.5-109.1)	105.6(103.5-107.3)	0.453[b]
Ca^{2+},mmol/L	2.19±0.02	2.33±0.03	0.000[a]
ALT,U/L	10(7-16)	11(7-16)	0.412[b]
AST,U/L	14(11-18)	15(12-18)	0.134[b]
Total protein,g/L	61.80±0.89	65.80±0.66	0.000[a]
Albumin,g/L	36.04±0.65	39.08±0.53	0.000[a]
Globulin,g/L	25.97±0.48	26.30±0.47	0.632[a]
Cholesterol,mmol/L	4.57±0.14	4.69±0.14	0.539[a]
Uric acid,μmol/L	390.19±11.37	381.81±10.13	0.583[a]
Urinary red cell,/μL	10.4(4.3-29.4)	8.1(3.9-31.1)	0.616[b]
Urinary white cell,/μL	6.6(2.7-11.5)	3.9(2.2-9.9)	0.146[b]
Urinary cast,/,μL	0.1(0-0.4)	0.1(0-0.4)	0.811[b]
NRP1 high,n%	73/115(63.5%)	46/102(45.1%)	0.007[c]

The bold values indicate $P < 0.05$.

Data are presented as mean ± SEM or median (25–75th percentiles) or a percentage.

Tubular NRP1 high cell was defined as the number of NRP1 positive cells ≥50th percentiles.

[a]t-test, [b]Mann–Whitney U test, [c]Pearson's chi-squared test.

All statistical analyses were two-sided.

Table S2. Sequence for Nrp1 Used for Fluorescence in Situ Hybridization (Related to methods section)

Gene	Sequence(5'to3')
	GTAACCGGGAGATGTGAGGTACCC
	AGGATTCGAGTCTTGCTCCAGGTC
Nrp1	TGGATAGAACGCCTGAAGAGGAGC
	TGTGGCTCTCTCAGGGTAGATCCT
	CCAGAAGGTCATACAGTGGGCAGA

Table S3. List and sequence for primers used for RT-qPCR(Related to methods section)

Gene	Forward(5'to3')	Reverse(5'to3')
Gapdh	TGACCTCAACTACATGGTCTACA	CTTCCCATTCTCGGCCTTG
Nrp1	CAAGGAGTGGCACAGCATCT	GGAGCACCACATCCACAGAA
Tgfbr1	TCTGCATTGCACTTATGCTGA	AAAGGGCGATCTAGTGATGGA
Havcr1	ACATATCGTGGAATCACAACGAC	ACTGCTCTTCTGATAGGTGACA
Lcn2	GCAGGTGGTACGTTGTGGG	CTCTTGTAGCTCATAGATGGTGC
Acta2	CCCAGACATCAGGGAGTAATGG	TCTATCGGATACTTCAGCGTCA
Pdgfrb	AGGAGTGATACCAGCTTTAGTCC	CCGAGCAGGTCAGAACAAAGG
Col1a1	GCTCCTCTTAGGGGCCACT	CCACGTCTCACCATTGGGG
Col1a2	TCGTGCCTAGCAACATGCC	TTTGTCAGAATACTGAGCAGCAA
Fn1	GCTCAGCAAATCGTGCAGC	CTAGGTAGGTCCGTTCCCACT
Nfkb1	ATGGCAGACGATGATCCCTAC	CGGAATCGAAATCCCCTCTGTT
Smad3	CATTCCATTCCCGAGAACACTAA	GCTGTGGTTCATCTGGTGGT
Ndufa4	TCCCAGCTTGATTCCTCTCTT	GGGTTGTTCTTTCTGTCCCAG
Sdhd	TGGTCAGACCCGCTTATGTG	GGTCCAGTGGAGAGATGCAG
Uqcrb	GGCCGATCTGCTGTTTCAG	CATCTCGCATTAACCCCAGTT
Cox7b	TTGCCCTTAGCCAAAAACGC	TCATGGAAACTAGGTGCCCTC
Atp5o	TCTCGACAGGTTCGGAGCTT	TTGACGGTGCGCTTGATGTAG
Pdhb	AAGAGGCGTTTTCACCGCTC	GTCACCGTATTTCTTCCACAGG
Acly	ACCCTTTCACTGGGGATCACA	GACAGGGATCAGGATTTCCTTG
Oghd	GTTTCTTCAAACGTGGGGTTCT	GCATGATTCCAGGGGTCTCAAA
Idh3a	TGGGTGTCCAAGGTCTCTC	CTCCCACTGAATAGGTGCTTTG

References

1. https://singlecell.broadinstitute.org/.
2. https://www.spatialomics.org/SpatialDB
3. https://www.biorender.com
