# Supplementary material for: Targeting the transmembrane cytokine co-receptor neuropilin-1 in distal tubules improves renal injury and fibrosis
Source: Nat Commun. 2024 Jul 8;15:5731. doi: 10.1038/s41467-024-50121-6 (PMC11231174; doi:10.1038/s41467-024-50121-6)
Supplement: Supplementary file 3 — Reporting Summary [file 41467_2024_50121_MOESM3_ESM.pdf]

nature portfolio

Corresponding author(s): Ying Yao, Gang Xu, Rui Zeng

Last updated by author(s): Jun 3, 2024

Reporting Summary

Nature Portfolio wishes to improve the reproducibility of the work that we publish. This form provides structure for consistency and transparency in reporting. For further information on Nature Portfolio policies, see our Editorial Policies and the Editorial Policy Checklist.

Statistics

For all statistical analyses, confirm that the following items are present in the figure legend, table legend, main text, or Methods section.

n/a	Confirmed	
☐	☒	The exact sample size (*n*) for each experimental group/condition, given as a discrete number and unit of measurement
☐	☒	A statement on whether measurements were taken from distinct samples or whether the same sample was measured repeatedly
☐	☒	The statistical test(s) used AND whether they are one- or two-sided<br>*Only common tests should be described solely by name; describe more complex techniques in the Methods section.*
☐	☒	A description of all covariates tested
☐	☒	A description of any assumptions or corrections, such as tests of normality and adjustment for multiple comparisons
☐	☒	A full description of the statistical parameters including central tendency (e.g. means) or other basic estimates (e.g. regression coefficient) AND variation (e.g. standard deviation) or associated estimates of uncertainty (e.g. confidence intervals)
☐	☒	For null hypothesis testing, the test statistic (e.g. *F*, *t*, *r*) with confidence intervals, effect sizes, degrees of freedom and *P* value noted<br>*Give P values as exact values whenever suitable.*
☒	☐	For Bayesian analysis, information on the choice of priors and Markov chain Monte Carlo settings
☒	☐	For hierarchical and complex designs, identification of the appropriate level for tests and full reporting of outcomes
☐	☒	Estimates of effect sizes (e.g. Cohen's *d*, Pearson's *r*), indicating how they were calculated

Our web collection on statistics for biologists contains articles on many of the points above.

Software and code

Policy information about availability of computer code

Data collection	There is no software was used for data collection
Data analysis	Single-cell data were analyzed using R 4.2.1, the other statistical analyses were analyzed GraphPad Prism software version 8.0.2 or IBM SPSS Statistics 26.

For manuscripts utilizing custom algorithms or software that are central to the research but not yet described in published literature, software must be made available to editors and reviewers. We strongly encourage code deposition in a community repository (e.g. GitHub). See the Nature Portfolio guidelines for submitting code & software for further information.

Data

Policy information about availability of data

All manuscripts must include a data availability statement. This statement should provide the following information, where applicable:

- Accession codes, unique identifiers, or web links for publicly available datasets
- A description of any restrictions on data availability
- For clinical datasets or third party data, please ensure that the statement adheres to our policy

The raw bulk RNA-seq data reported in this paper have been deposited in the Genome Sequence Archive63 in National Genomics Data Center64, China National Center for Bioinformation / Beijing Institute of Genomics, Chinese Academy of Sciences (GSA: CRA016479 [https://bigd.big.ac.cn/gsa/browse/CRA016479]).
The pTECs proteomics data have been deposited to the ProteomeXchange Consortium via the PRIDE65 partner repository with the dataset identifier PXD052244

[http://www.ebi.ac.uk/pride/archive/projects/PXD052244].
The kidney proteomics data and modified proteomics detected the changes in Kcr have been deposited to the ProteomeXchange Consortium via the PRIDE65 partner repository with the dataset identifier PXD052293 [http://www.ebi.ac.uk/pride/archive/projects/PXD052293].
The scRNA-seq data used in this study are available in Figshare (DOI: http://doi.org/10.6084/m9.figshare.25845748). Source data are provided with this paper.

Research involving human participants, their data, or biological material

Policy information about studies with human participants or human data. See also policy information about sex, gender (identity/presentation), and sexual orientation and race, ethnicity and racism.

Reporting on sex and gender	YES
Reporting on race, ethnicity, or other socially relevant groupings	Not included
Population characteristics	217 patients were included, with an average age of 41.72 ± 1.039 for patients with eGFR<30ml/min 1.73m2, and 40.11 ± 1.14 for patients with eGFR≥30ml/min 1.73m2. All patients were renal dysfunction after kidney transplantation. Detailed information can be found in Supplementary File Table S1.
Recruitment	The patients were randomly selected from Tongji Hospital, Tongji Medical College, Huazhong University of Science and Technology, Wuhan , China, there would no any self-selection bias.
Ethics oversight	The institutional ethical committee board approved the clinical protocol. Human tissue samples were obtained from individuals who provided informed consent. The research was performed according to the Helsinki's declaration principles.

Note that full information on the approval of the study protocol must also be provided in the manuscript.

Field-specific reporting

Please select the one below that is the best fit for your research. If you are not sure, read the appropriate sections before making your selection.

☒ Life sciences ☐ Behavioural & social sciences ☐ Ecological, evolutionary & environmental sciences

For a reference copy of the document with all sections, see nature.com/documents/nr-reporting-summary-flat.pdf

Life sciences study design

All studies must disclose on these points even when the disclosure is negative.

Sample size	The sample sizes were determined based on pilot experiments and previous experience with the models and the methods used in this study: PMID: 34270930. We chosed 6 or more animals mostly.
Data exclusions	No data excluded.
Replication	The experiments were independently repeated three times.
Randomization	All experiments are randomized.
Blinding	All experiments are blinding.

Reporting for specific materials, systems and methods

We require information from authors about some types of materials, experimental systems and methods used in many studies. Here, indicate whether each material, system or method listed is relevant to your study. If you are not sure if a list item applies to your research, read the appropriate section before selecting a response.

Materials & experimental systems

n/a	Involved in the study	
☐	☒	Antibodies
☒	☐	Eukaryotic cell lines
☒	☐	Palaeontology and archaeology
☐	☒	Animals and other organisms
☒	☐	Clinical data
☒	☐	Dual use research of concern
☒	☐	Plants

Methods

n/a	Involved in the study	
☒	☐	ChIP-seq
☒	☐	Flow cytometry
☒	☐	MRI-based neuroimaging

Antibodies

Antibodies used	Kim1 (1:1000, R&D Systems, USA, AF1817),Nrp1 (1:100, Abcam, UK, ab81321),Nrp1 (1:100, R&D Systems, USA, FAB5994P),Nrp1 (1:100, Santa Cruz Biotechnology, USA, sc-5307) ,S12a3 (1:100, Abcam, UK, ab95302),Tgfr1 (1:100, Proteintech, China, 30117-1-AP), Tnr1a (1:100, Abcam, UK, ab223352),Cdh16 (1:100, Proteintech, China,15107-1-AP),Cox4i1 (1:1000, CST, 4850),Nfkb1 (1:1000, CST, 13586),Smad3 (CST, 1: 1000, 9523),Pdgfrb (1:1000, Abcam, UK, ab69506),α-SMA (1:1000, Abcam, UK,ab7817) ,β-actin (1:1000, CST, 4967)<br>Anti- Crotonyllysine(PTM-502), Anti- 2-Hydroxyisobutyryllysine(PTM-802), Anti- Succinyllysine (PTM-419), Anti- Acetyllysine (PTM-105RM), Anti- β-Hydroxybutyryllysine(PTM-1201RM), Anti- Lactyl Lysine(PTM-1401RM), and Anti- Malonyllysine(PTM-902) (1:1000) were provided by Jingjie PTM BioLab.
Validation	Antibodies were validated by the manufacturers as statedon their websites.described on the following websites:<br>Kim1 (R&D Systems, AF1817, https://www.rndsystems.com/cn/products/mouse-tim-1-kim-1-havcr-antibody_af1817),Nrp1 (Abcam, ab81321, https://www.abcam.cn/products/primary-antibodies/neuropilin-1-antibody-epr3113-ab81321.html),Nrp1 (Santa Cruz Biotechnology, sc-5307, https://www.scbt.com/zh/p/neuropilin-antibody-a-12),S12a3 (Abcam, ab95302, https://www.abcam.cn/products/primary-antibodies/slc12a3-antibody-ab95302.html),Tgfr1 (Proteintech, 30117-1-AP, https://www.ptgcn.com/products/TGFBR1-Antibody-30117-1-AP.htm#product-information),Tnr1a (Abcam, UK, ab223352, https://www.abcam.cn/products/primary-antibodies/tnf-receptor-i-antibody-ab223352.html),Cdh16 (Proteintech, 15107-1-AP, https://www.ptgcn.com/products/CDH16-Antibody-15107-1-AP.html),Cox4i1 (CST, 4850, https://www.cellsignal.cn/products/primary-antibodies/cox-iv-3e11-rabbit-mab/4850),Nfkb1 (CST, 13586, https://www.cellsignal.cn/browse/?tab=product&search=Nfkb1&site-search-type=Products),Smad3 (CST, 9523, https://www.cellsignal.cn/products/primary-antibodies/smad3-c67h9-rabbit-mab/9523),Pdgfrb (Abcam, ab69506, https://www.abcam.cn/products/primary-antibodies/pdgfr-beta-antibody-42g12-ab69506.html),α-SMA (Abcam,ab7817, https://www.abcam.cn/products/primary-antibodies/alpha-smooth-muscle-actin-antibody-1a4-ab7817.html),β-actin (CST, 4967, https://www.cellsignal.cn/products/primary-antibodies/b-actin-antibody/4967),Anti- Crotonyllysine(Jingjie PTM BioLab, PTM-502, http://www.ptm-biolab.com.cn/productDetail.html?id=4759), Anti- 2-Hydroxyisobutyryllysine(Jingjie PTM BioLab, PTM-802, http://www.ptm-biolab.com.cn/productDetail.html?id=4611), Anti- Succinyllysine (Jingjie PTM BioLab, PTM-419, http://www.ptm-biolab.com.cn/productDetail.html?id=4758), Anti- Acetyllysine(Jingjie PTM BioLab, PTM-105RM, http://www.ptm-biolab.com.cn/productDetail.html?id=6244), Anti- β-Hydroxybutyryllysine(Jingjie PTM BioLab, PTM-1201RM, http://www.ptm-biolab.com.cn/productDetail.html?id=4599), Anti- Lactyl Lysine(Jingjie PTM BioLab, PTM-1401RM, http://www.ptm-biolab.com.cn/productDetail.html?id=5863), Anti- Malonyllysine(Jingjie PTM BioLab, PTM-902, http://www.ptm-biolab.com.cn/productDetail.html?id=4595)

Animals and other research organisms

Policy information about studies involving animals; ARRIVE guidelines recommended for reporting animal research, and Sex and Gender in Research

Laboratory animals	C57BL/6J mice (6-8week);Ksp icre / Nrp1 f/+ mice(6-8week);Tgfbr1 fl/+,Ksp-icre mice(6-8week);Nrp1/Tgfbr1 fl/+,Ksp-icre mice (6-8week);Nrp1 fl/+,Col1a2-icre mice(6-8week);Nrp1 fl/+,Pdgfrb-icre mice (6-8week). The housing conditions for the mice has been described in the manuscript.All mice were housed in a specific pathogen-free and sterile environment at the Animal Experimental Center of Tongji Medical College, Huazhong University of Science and Technology. All mice were fed with normal chow diet (about 4.2% fat and 21% protein, WQJX Bio-Technology). The mice were maintained at 22°C temperature and 50% humidity conditions, following a 12-hour light-dark cycle. C57BL/6 mice underwent a one-week acclimation period prior to the experiments. The housing and experimental procedures for the mice strictly adhered to the guidelines of the National Institutes of Health (NIH) and the Animal Management Committee of Tongji Medical College, Huazhong University of Science and Technology. All mice used in this study were males, aged 8-12 weeks. All mice were assigned to groups randomly.
Wild animals	No wild animals
Reporting on sex	YES
Field-collected samples	NO
Ethics oversight	Animal care and experimental procedures were approved by the Animal Ethics Committee of Huazhong University of Science and Technology ([2023] IACUC number: 3181).

Note that full information on the approval of the study protocol must also be provided in the manuscript.

Plants

Seed stocks	*Report on the source of all seed stocks or other plant material used. If applicable, state the seed stock centre and catalogue number. If plant specimens were collected from the field, describe the collection location, date and sampling procedures.*
Novel plant genotypes	*Describe the methods by which all novel plant genotypes were produced. This includes those generated by transgenic approaches, gene editing, chemical/radiation-based mutagenesis and hybridization. For transgenic lines, describe the transformation method, the number of independent lines analyzed and the generation upon which experiments were performed. For gene-edited lines, describe the editor used, the endogenous sequence targeted for editing, the targeting guide RNA sequence (if applicable) and how the editor was applied.*
Authentication	*Describe any authentication procedures for each seed stock used or novel genotype generated. Describe any experiments used to assess the effect of a mutation and, where applicable, how potential secondary effects (e.g. second site T-DNA insertions, mosiacism, off-target gene editing) were examined.*
